# Carcinosarcoma, a Rare Malignant Neoplasm of the Pancreas

**DOI:** 10.3390/curroncol28060442

**Published:** 2021-12-12

**Authors:** Jaffar Khan, Liang Cheng, Michael G. House, Shunhua Guo

**Affiliations:** 1 Department of Pathology and Laboratory Medicine, Indiana University School of Medicine, Indianapolis, IN 46202, USA; khanja@iu.edu (J.K.); lcheng@iupui.edu (L.C.); 2 Department of Surgery, Indiana University School of Medicine, Indianapolis, IN 46202, USA; mhouse3@iuhealth.org

**Keywords:** pancreas, malignant neoplasm, biphasic, carcinosarcoma, pancreatic ductal carcinoma

## Abstract

Carcinosarcoma of the pancreas is a rare entity with poor prognosis. Here, we report a case of pancreatic carcinosarcoma in a 68-year-old male patient who underwent a pancreatoduodenectomy for a unilocular cystic mass in the head of the pancreas. Histologically, the lesion showed a biphasic tumor with a carcinoma component and a spindle cell sarcomatous component, which were intimately intermingled. Most of the carcinoma components are well-differentiated ductal adenocarcinoma with small areas of moderately to poorly differentiated ductal adenocarcinoma. The sarcomatous component is a high-grade highly cellular spindle cell tumor with frequent mitosis and apoptosis. Immunohistochemical studies demonstrated that the carcinomatous component was positive for epithelial markers and cyclin D1, and the sarcomatous component was negative for these markers while positive for vimentin, p16, and DOG1 with patchy positivity for S100. Other markers, including SOX10, CD117, Melan A, HMB45, actin, desmin, myogenin, beta-catenin, TLE1, and p53, were negative in both components. Molecular studies demonstrated that the tumor was microsatellite stable. Whole exome next generation sequencing analysis was performed and no pathogenic alterations in the genes were identified.

## 1. Introduction

Carcinosarcoma of the pancreas is a rare tumor with limited clinical and pathologic data reported in the literature [1]. As per the World Health Organization (WHO) classification of tumors of the digestive system, the carcinosarcoma of the pancreas is classified together with sarcomatoid carcinoma and anaplastic carcinomas under the category of undifferentiated carcinoma of the pancreas [2]. Carcinosarcoma of the pancreas have a component that is epithelial in origin, which is usually an adenocarcinoma and a spindle cell sarcomatous component. Each of these elements show distinct morphological and immunohistochemical features [3]. The treatment of choice for most reported cases is surgery. Prognosis is similar or even worse than that of classic pancreatic ductal adenocarcinoma [1,4]. Here, we present a case of pancreatic carcinosarcoma, including the clinical and histopathological features and the results of immunohistochemical and molecular studies.

## 2. Case Report

A 68-year-old male with a history of diabetes was admitted to our hospital with a two-week history of abdominal pain, jaundice, nausea, anorexia, and episodes of loose stools. Physical examination revealed right-sided abdominal tenderness. Laboratory examination revealed slightly higher bilirubin levels (0.4 mg/dL), but serum amylase and lipase levels, and complete blood count were all within the normal range. Abdominal computed tomography demonstrated a large cystic mass in the head of the pancreas, which measured 8.1 × 7.5 × 7.4 cm, and dilatation of the common bile duct, measuring 22 mm in diameter. There was also dilatation of the pancreatic duct, measuring 5 mm in diameter. The remainder of the pancreas was grossly unremarkable. Fine needle aspiration (FNA) was performed using endoscopic ultrasound (EUS). The EUS FNA fluid test showed a CEA level > 900 ng/mL, and fluid cytology was negative for malignancy or high-grade dysplasia. Endoscopic retrograde cholangiopancreatography (ERCP) was performed with biliary stent placement, which led to the resolution of his jaundice. An extended pylorus-sparing pancreaticoduodenectomy was performed. The operation was uneventful, and the patient was discharged 4 days after surgery.

Gross examination: The pancreatic head was entirely replaced by a mass lesion measuring 8.2 × 7.9 × 7.2 cm and was a unilocular cystic lesion containing gray-green turbid fluid with granular material. The cyst structure appeared to communicate with both the main and side duct branches. The cyst lining was gray-green to yellow, trabecular, and glistening to granular with few fibrous strands that arborized through the cystic structure and anchored at opposing sides of the cyst. Using a standard pancreatic cancer sampling protocol, paraffin-embedded sections of formalin-fixed tissue were studied by routine histology at the Indiana University Pathology Laboratory.

**Figure 1:**
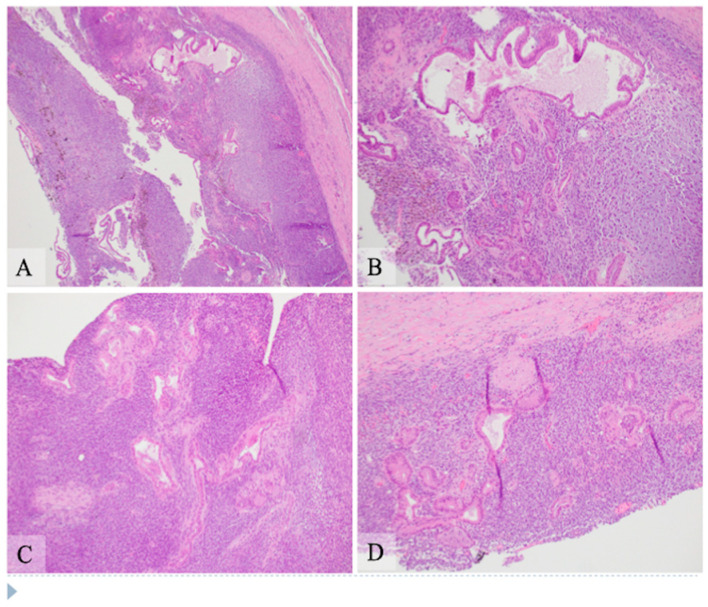
Hematoxylin and eosin stain shows pancreatic carcinosarcoma with the well differentiated epithelial component and the spindle cell component. The left lower corner of panels (**A**,**B**), the left upper corner of panel (**C**) and the bottom portion of panel (**D**) are the cystic areas. (**A**) Low power view (original magnification 40×). Majority of the cyst wall is composed of sheets of spindle cells with scattered epithelial component with small or large duct structures. There are foci of hemosiderin deposition, mostly in the spindle cell areas, suggestive of previous hemorrhage. (**B**) Intermediate power view (original magnification 100×) of an upper area in panel **A**. The ducts are lined by a single layer of columnar cells with basally located round nuclei with minimal atypia and moderate amount of eosinophilic cytoplasm without mucinous contents. (**C**,**D**) Additional intermediate power view (original magnification 100×) of areas with well differentiated ductal adenocarcinoma closely intermixed with the spindle cell componentt. The epithelium of the ducts shows minimal atypia and moderate amount of eosinophilic cytoplasm. The spindle cells intimately surround the epithelial component with hyperchromatic and elongated nuclei and scant amount of cytoplasm.

**Figure 2:**
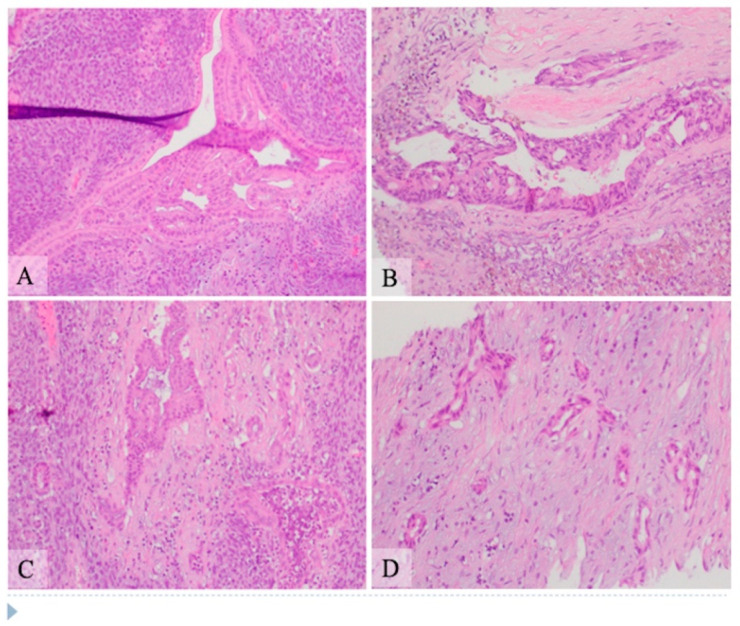
Hematoxylin and eosin stain shows pancreatic carcinosarcoma with moderately differentiated ductal adenocarcinoma and the spindle cell component, intermediate power view (**A**–**D**, original magnification 200×). (**A**) The epithelial component shows complex structure with convoluted glandular structures which has single layer of cells with moderate amount of eosinophilic cytoplasm and mildly enlarged nuclei. (**B**) The epithelial component shows multilayers of cells with cribriform-type interconnection. The nuclei of cells are moderately enlarged, hyperchromatic and irregular. (**C**) A duct (upper middle area) with simple to multilayered epithelial cells and adjacent scattered smaller and irregular ducts with hyperchromatic nuclei. The duct in the right lower corner has focal necrosis with inflammatory cells in the lumen. (**D**) Some areas of the tumor show small and angulated ducts embedded in a desmoplastic stromal background, simulating the typical morphology of a conventional pancreatic ductal carcinoma.

**Figure 3:**
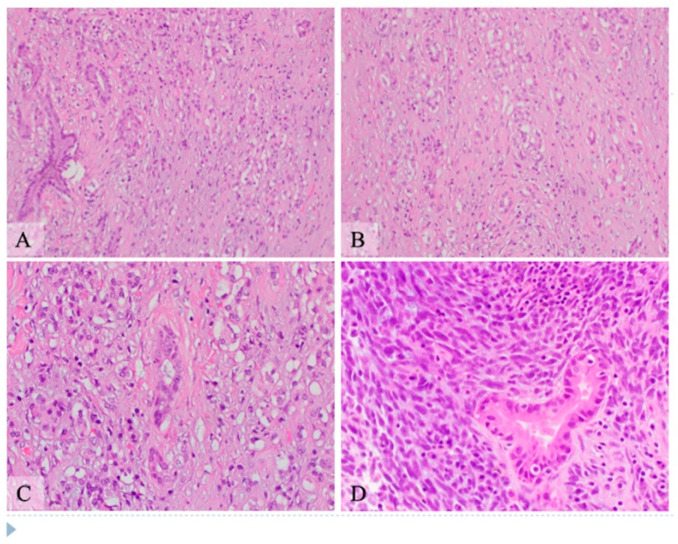
Hematoxylin and eosin stain shows pancreatic carcinosarcoma with poorly differentiated ductal adenocarcinoma and spindle cell component, intermediate and high power view ((**A**,**B**) original magnification 200×, (**C**,**D**) original magnification 400×). (**A**) Well to moderately differentiated ductal structures (left side) with transition to poorly formed ducts and to small sheets of epithelioid cells with no apparent structure. (**B**) Scattered small and poorly formed ductal structures are intermixed with infiltrative small sheets of single cells. (**C**) Small to poorly formed ducts (central area) are surrounded by small nests to single cells with vesicular nuclei, prominent nucleoli and small amount of eosinophilic to clear cytoplasm. (**D**) An atypical duct with surrounding spindle cells component. The ductal epithelium has hyperchromatic nuclei, some of which show conspicuous nucleoli. The spindle cell component is highly cellular with compact elongated cells with hyperchromatic nuclei and scant cytoplasm. Mitosis and apoptosis are present in the spindle cell area.

Microscopic examination: Histologically, the tumor showed two components composed of an epithelial component and a spindle cell component that were intimately intermingled together. The epithelial component had features ranging from well differentiated to moderately and poorly differentiated pancreatic ductal adenocarcinoma. The majority of the epithelial component was well differentiated with simple small to large ductal structures lined by a single layer of columnar to cuboidal cells, which had small and basally located nuclei with smooth and round nuclear contours and open chromatin. They had a moderate amount of eosinophilic cytoplasm without mucinous content (Figure 1). The moderately differentiated component showed a more complex glandular structure with convoluted and interconnected ducts with a single layer of cells or a cribriform-type structure including multiple layers of cells with enlarged and irregular nuclei (Figure 2). Some areas showed prototypical morphology of conventional pancreatic ductal carcinoma with small and angulated ducts infiltrating the desmoplastic stroma. The poorly differentiated epithelial component was small and focal. It showed vague and poorly formed ductal structures, or solid nests to small sheets of dispersed epithelioid cells with no ductal structures (Figure 3). These cells had enlarged vesicular nuclei with irregular nuclear contours and conspicuous nucleoli. The spindle cell component was highly cellular with compact spindle cells, which showed hyperchromatic and elongated nuclei with scant cytoplasm. There was rare mitosis in the epithelial component, but the spindle cell component showed frequent mitosis with up to 12 mitoses per 10 high-power fields. Frequent apoptosis was also observed in spindle cell areas. Scattered necrotic areas were present in both components. There were no osteoclast-like giant cells or rhabdomyoblasts and no osteoid formation. There were foci of hemosiderin deposition, especially in the spindle cell areas surrounding the cystic lining. None of the ducts showed papillary or mucinous features. No areas subjacent to the epithelial component showed ovarian stroma-like features. All margins were negative for tumor. Twenty lymph nodes were present, all of which were negative for metastatic tumors. The pathologic staging was pT3pN0.

**Figure 4:**
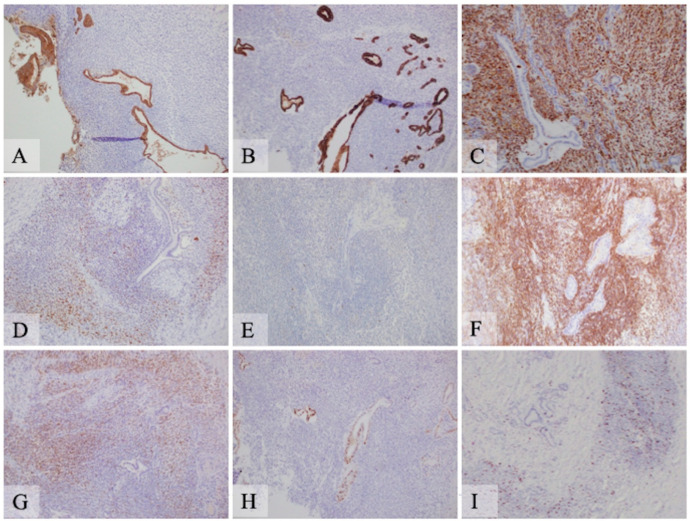
Immunohistochemical analysis of pancreatic carcinosarcoma (original magnification 200×. (**A**) Pancytokeratin AE1/AE3 highlights the epithelial component, but the spindle cell component is negative. (**B**) CK19 is positive in the ductal epithelial cells and is negative in the spindle cell component. (**C**) Vimentin is strongly and diffusely positive in the spindle cell component but is negative in the epithelial component. (**D**) S100 shows patchy positivity in the spindle cell component but not in the epithelial component. (**E**) SOX10 is negative in both the spindle cell and epithelial components. (**F**) DOG1 shows strong and diffuse positivity in the spindle cell component but not in the epithelial component. (**G**) p16 is positive in the spindle cell component and negative in the epithelial components. (**H**) Cyclin D1 shows patchy positivity in the epithelial component but is negative in the spindle cell component. (**I**) Ki-67 demonstrates high level of nuclear staining (about 20%) in the spindle cell component with low level staining (about 2%) in the epithelial component.

Immunohistochemistry: Extensive immunohistochemical studies were performed at the Indiana University Pathology Laboratory due to the mixed features of the lesion (Figure 4). The epithelial component was positive for markers of pancytokeratin AE1/AE3, epithelial membrane antigen (EMA), CK7, and CK19, and negative for MUC2, MUC5, MUC6, synaptophysin, and chromogranin. Spindle cells were negative for these markers. The spindle cells were diffusely positive for vimentin and DOG1 with patchy positivity for S100. Both epithelial and spindle tumor cells were negative for the estrogen receptor, CD10, inhibin, TLE1, SOX10, Melan A, HMB45, actin, desmin, myogenin, MyoD1, STAT6, and CD117. No nuclear staining was observed for β-catenin. CD163 highlighted cells with hemosiderin deposition, consistent with histiocytes. The tumor cells were negative for CD21 and CD35 expression. P53 showed a wild type staining pattern with no complete loss or overexpression in tumor cells of both components. Cyclin D1 showed patchy nuclear staining in the epithelial component but was negative in the spindle cell component. P16 was positive in the spindle cell component but negative in the epithelial component. The spindle cells demonstrated approximately 20% positivity of Ki-67 nuclear staining, while it showed only scant (about 2%) nuclear staining in the epithelial component (Table 1). Additional immunohistochemical staining for PDL-1 (SP142), MLH1, MSH2, MSH6, and PMS2 was performed at the Caris Life Science Laboratory (Phoenix, Arizona) and showed negativity (0%) for PDL-1 expression and intact protein expression of MLH1, MSH2, MSH6, and PMS2.

Molecular study: Molecular analysis of the tumor tissue was first performed by Indiana University Molecular Pathology Laboratory and showed that the tumor was microsatellite stable with no mutation in BRAF, KRAS, and NRAS genes. Additionally, the tumor tissue was sent to the Caris Life Science Laboratory (Phoenix, AZ, USA) for next generation sequencing analysis of whole exome sequencing (WES). Direct sequence analysis was performed on genomic DNA using Illumina NovaSeq 6000 sequencers. Tumor mutation burden (TMB) was low and genomic loss of heterozygosity (LOH) was also low, with 10% of the tested genomic segments exhibiting LOH. The whole exome sequencing in our case showed no pathogenic alterations in the genes, such as BRAF, ATM, BRCA1, BRCA2, PALB2, SMAD4, NRG1, and NTRK1/2/3. However, the results for AXL1, HDAC1, MED12, NOTCH1, PIK3CB, POLD2, PRKACA, PTPN11, TERT, and XRCC1 were indeterminate because of the low coverage of exons in these genes.

The patient was followed up for three months after surgical resection. The last time he had an appointment for discussing the adjuvant chemotherapy. But he was then lost to follow up without receiving adjuvant chemotherapy.

## 3. Discussion

Carcinosarcomas are rare malignant neoplasms. They are histologically characterized by two distinct components: an epithelial component and a mesenchymal component. These dual malignancies most frequently arise in the uterus, [5,6], but they have also been reported in many other organs such as the ovaries, prostate, breast, urinary tract, lung, larynx, and parotid gland, as well as in the gastrointestinal system, such as in the esophagus, stomach, liver, gallbladder, duodenum, and pancreas. The most recent (5th edition) World Health Organization classification of tumors of the exocrine pancreas lists it under the section of undifferentiated carcinoma, a variant of pancreatic ductal adenocarcinoma, indicating the commonly held concept that the sarcomatous component is likely derived from the carcinoma component [2,3]. Previous studies suggested that most pancreatic carcinosarcomas appear to be of monoclonal origin and seem to arise from a carcinoma via metaplastic transformation of a subclone of the tumor, probably by the epithelial–mesenchymal transition mechanism [3,7]. In one report, clonality of the two distinct tumor components was assessed by microdissection with subsequent genetic analysis for KRAS and TP53 gene mutation and showed the same genetic alterations in the carcinomatous and the sarcomatous components, strongly suggesting a common origin [8]. Another study demonstrated that an identical mutation (G to A transition) at exon 2 of the KRAS gene was detected in both the carcinomatous and sarcomatous components [9]. However, molecular studies in our case showed that pathogenic mutations were not detected in this tumor, and the tumor showed low tumor mutation burden and low loss of heterozygosity with intact microsatellite stability. Therefore, the pathogenesis of this tumor remains unknown.

Histologically, the tumor in our case showed that the carcinoma and sarcomatous components were intimately intermixed. Most of the carcinoma was well-differentiated ductal adenocarcinoma, with well-formed ducts and bland-appearing epithelial cells. Small scattered areas showed moderately differentiated ductal adenocarcinoma with more complex ductal structures. Small areas showed poorly differentiated epithelial components with infiltrating atypical epithelioid cells, forming small sheets, nests, or single cells. The spindle cell component was highly cellular, with frequent mitosis and apoptosis. These findings suggest a progressive evolution/dedifferentiation of a carcinoma from low-grade to moderate- and high-grade carcinoma and then transition/transformation to a high-grade spindle cell sarcomatous tumor, likely through the mechanism of so-called epithelial–mesenchymal transition. There were foci of hemosiderin deposition suggestive of a previous hemorrhage. It is likely that multiple episodes of hemorrhage and/or necrosis resulted in central cystic formation, as observed radiologically and grossly. No areas showed papillary structures or cells with mucinous features. Intraductal pancreatic mucinous neoplasm (IPMN) often shows positivity for MUC5 and MUC6 in the gastric and pancreaticobiliary types and positivity for MUC2 and MUC5 in the intestinal type, but these markers were all negative in this tumor. Therefore, there was no evidence to suggest that this tumor developed from an IPMN. This was a male patient, and there was no ovarian-type stroma underlying the epithelium. The tumor was negative for the estrogen receptor and inhibin. Hence, there was no evidence to suggest that this tumor was derived from a mucinous cystic neoplasm.

Immunohistochemistry studies showed that the adenocarcinoma cells had a positive reaction for epithelial markers, as expected, while no staining of these markers was observed in the sarcomatous component. Carcinosarcoma can be differentiated from sarcomatoid carcinoma which usually shows focal cytokeratin reactivity in the spindle cell component. The spindle cell component of this tumor was positive for DOG1 but negative for CD117. DOG1 (Discovered on GIST-1) is not a specific marker for gastrointestinal stromal tumors (GIST), for it can also be positive in carcinomas of salivary or sweat glands and in sarcomas such as malignant peripheral nerve tumors, desmoplastic/spindle cell melanoma, and prostatic stromal sarcoma [10,11]. Even though there is patchy S100 reactivity in the spindle cell component, SOX10 is completely negative. With the close juxtaposition of the low- to high-grade ductal carcinoma with the spindle cell component, the morphology is not consistent with a malignant peripheral nerve tumor. With the negativity of SOX10, Melan A, and HMB45, it does not support desmoplastic/spindle cell melanoma. The presence of both epithelial and spindle cell components raises the possibility of biphasic synovial sarcoma. However, it is very unusual for the epithelial component to demonstrate a spectrum of well, moderately, and poorly differentiated carcinoma in synovial sarcoma. TLE1 was also completely negative in both the components. The spindle cells were negative for actin, desmin, Myod1, and myogenin, excluding leiomyosarcoma or rhabdomyosarcoma, and they were also negative for CD21 and CD35, excluding follicular dendritic sarcoma.

In our case, the epithelial component was positive for cyclin D1 and negative for p16, while the sarcomatous component was the opposite. The P53 stain pattern was the wild type, with no loss of expression or overexpression in both components. It is known that cell cycle-regulating molecules are essential for the control of cellular proliferation. Two pathways are involved in cell cycle modulation: the p16-cyclin D1/CDK4-pRb pathway and the p53 pathway [12]. Amplification of the cyclin D1 gene CCND1 or its overexpression has been reported in multiple carcinomas. Activation of cyclin D1/cyclin-dependent kinase CDK4/6 complex leads to the transition of the cell cycle from the G to S phase, which may be related to tumorigenesis. P16 is a tumor suppressor gene, and the mechanisms of its overexpression in HPV-independent tumors are complicated, some of which can be related to deregulation of the Rb (retinoblastoma) gene [13]. It may be postulated that cyclin D1 overexpression can be one of the events associated with the transformation of the benign duct to dysplasia or progression of dysplasia to adenocarcinoma, and p16 overexpression may be related to the progression to high-grade sarcoma.

For pancreatic carcinosarcoma, available data are limited regarding its treatment and prognosis [1,2]. The largest case series reported on a National Cancer Institute database from 1976 to 2016 showed a median overall survival of 6 months [4]. However, some cases with long-term survival have been reported [14,15]. Surgical resection is the best option for patients with pancreatic carcinosarcoma when it is resectable. Systemic chemotherapy is indicated for patients with metastasis or other contraindications for surgery. Despite surgery and adjuvant chemotherapy, the recurrence rate is high, and the prognosis is poor. However, there are no standard chemotherapy recommendations for first-line, adjuvant, or additive therapy settings because of the limited number of cases studied. The two components of this tumor may show different sensitivities to chemotherapy, and, therefore, chemotherapy for conventional pancreatic ductal carcinoma may not be the best choice [16].

## 4. Conclusions

In summary, this case highlights the morphology, immunohistochemical characteristics, and molecular study results of pancreatic carcinosarcoma, a rare entity of malignant pancreatic neoplasm. The presence of well to moderately and poorly differentiated ductal adenocarcinoma in close contiguity with a high-grade spindle cell sarcomatous component implies the progressive evolution of the tumor. Molecular studies showed that the tumor was microsatellite stable, and no pathogenic mutation was identified in genes by whole exome sequencing. More studies on this rare but ominous tumor are needed to understand its pathogenesis, treatment, and prognosis.

## Figures and Tables

**Table 1:** Immunohistochemical stain results.

Markers	Adenocarcinoma	Sarcoma
CK AE1/3	Positive	Negative
EMA	Positive	Negative
p53	Negative	Negative
CK7	Positive	Negative
MUC2	Negative	Negative
MUC5	Negative	Negative
MUC6	Negative	Negative
ER	Negative	Negative
CD117	Negative	Negative
CD10	Negative	Negative
Desmin	Negative	Negative
S-100	Negative	Focal positive
Inhibin	Negative	Negative
TLE	Negative	Negative
Sox-10	Negative	Negative
Melan A	Negative	Negative
HMB-45	Negative	Negative
Actin	Negative	Negative
Myogenin	Negative	Negative
MyoD-1	Negative	Negative
STAT6	Negative	Negative
DOG1	Negative	Positive
Beta-catenin	Negative	Focal cytoplasmic
Ki67	2%	20%

## Data Availability

Not applicable.
